# Genome-Wide Scans for Selection Signatures in Ningxia Angus Cattle Reveal Genetic Variants Associated with Economic and Adaptive Traits

**DOI:** 10.3390/ani15010058

**Published:** 2024-12-30

**Authors:** Haiqi Yin, Yuan Feng, Yu Wang, Qiufei Jiang, Juan Zhang, Jie Zhao, Yafei Chen, Yaxuan Wang, Ruiqi Peng, Yahui Wang, Tong Zhao, Caihong Zheng, Lingyang Xu, Xue Gao, Huijiang Gao, Junya Li, Zezhao Wang, Lupei Zhang

**Affiliations:** 1 State Key Laboratory of Animal Biotech Breeding, Institute of Animal Science, Chinese Academy of Agricultural Sciences, Beijing 100193, China; yinhaiqi127@163.com (H.Y.); wangyaxuan000@126.com (Y.W.); pengrq12138@163.com (R.P.); wang1434243198@163.com (Y.W.); zhaotong19981204@163.com (T.Z.); zhengcaihong@caas.cn (C.Z.); xulingyang@caas.cn (L.X.); gaoxue76@126.com (X.G.); gaohuijiang@caas.cn (H.G.); lijunya@caas.cn (J.L.); 2 Ningxia Autonomous Region Animal Husbandry Workstation, Yinchuan 750004, China; fyuan@126.com (Y.F.); wy_690831@163.com (Y.W.); jiangqiufei@163.com (Q.J.); zhaojie202412@163.com (J.Z.); 3 School of Animal Science and Technology, Ningxia University, Yinchuan 750021, China; zhangjkathy@126.com; 4 Yinchuan Animal Husbandry Technology Extension Service Center, Yinchuan 750021, China; cyf12022412@163.com

**Keywords:** Angus cattle, whole-genome resequencing, selection signatures, iHS, immune-related gene, economic trait

## Abstract

The genetic improvement of beef cattle breeds is crucial for the advancement of the beef cattle industry. Whole-genome resequencing technology has been widely applied in genetic breeding as well as research on selection signatures in beef cattle. In this study, 20× whole-genome resequencing was performed on 282 Angus cattle from the Ningxia region, and a high-quality dataset encompassing extensive genomic variations across the entire genome was constructed. The iHS test identified 495 selection signal regions, which included pregnancy-associated glycoprotein (*PAG*) family genes and immune-related genes such as UL16-binding protein 21 (*ULBP21*), CD1b molecule (*CD1B*), and tumor necrosis factor ligand superfamily member 11 (*TNFSF11*). A quantitative trait locus (QTL) enrichment analysis revealed that several economic traits, including longissimus muscle area, marbling score, carcass weight, average daily gain, and milk yield, were significantly enriched in cattle with these selection signatures. Although the enrichment of QTLs for health traits was low, immune-related genes may indirectly contribute to improvements in production performance. These findings show the genetic basis of economic and adaptive traits in Ningxia Angus cattle, providing a theoretical foundation and guidance for further genetic improvement and breeding strategies.

## Simple Summary

Angus cattle are highly valued by breeders due to their outstanding adaptability, superior meat quality, and excellent reproductive performance. In recent years, the Ningxia region has imported Angus cattle and frozen semen from Australia, the United States, and New Zealand. To better understand the genetic improvement in the selection history of the Ningxia Angus population, we performed whole-genome resequencing of 282 Angus cattle from the Ningxia region to identify selection signatures associated with improved productivity and adaptability in this study. These findings may provide valuable insights for future livestock breeding programs.

## 1. Introduction

The Aberdeen Angus breed is indigenous to the northeastern region of Scotland. Due to its strong adaptability, high meat quality, and low incidence of calving difficulties [1,2], it has become one of the most popular beef cattle breeds in the United Kingdom, Canada, and the United States. To improve the development of the beef cattle industry in Ningxia, 2251 Angus cows were introduced from Australia in 2014, followed by further introductions from New Zealand, Australia, and Uruguay in subsequent years. Additionally, Ningxia has been consistently importing 3–5 doses of frozen semen from Angus bulls in Argentina, the United States, and Canada every year to avoid inbreeding and improve the genetic diversity and herd quality. In 2024, the Angus population in Ningxia reached over 25,000. In line with the requirements for the high-quality development of the beef cattle industry, Ningxia has formulated a scientific breeding plan aimed at improving the beef yield, quality, and overall economic efficiency. The plan is based on imported purebred Angus cows and combines an open nucleus breeding system with scientific selection efforts to continuously optimize the genetic quality of the population. Moreover, the Ningxia region, with an average altitude of 1500–2200 m, an annual temperature of 6.2 °C, over 400 mm of annual precipitation, and abundant forage resources, offers the optimal environmental conditions for the growth and genetic improvement of Angus cattle. Utilizing confined feeding systems and total mixed ration (TMR) feeding models, combined with scientific breeding management, has not only improved the growth performance and meat quality of Angus cattle but also enhanced their adaptability to the local conditions. This has greatly enriched the genetic variation of the cattle population in Ningxia and accelerated the development of the regional beef cattle industry.

With the continuous development of second-generation sequencing technologies, whole-genome resequencing has become an essential tool for revealing the genetic basis of complex traits. Whole-genome resequencing can accurately detect genetic variants such as single nucleotide polymorphisms (SNPs) and insertions–deletions (indels), revealing genetic differences between individuals or populations [3]. Whole-genome resequencing also plays a critical role in population genetics research, enabling the analysis of genetic structures, gene flow, and natural selection [4,5], while also providing key insights into species evolution and environmental adaptation [6,7].

The processes of domestication, natural selection, and artificial selection all leave selection signatures within the genome. Analyzing these signals can reveal adaptation mechanisms and selection directions and help identify genes related to economic traits [8,9,10]. To date, a plethora of methods have been proposed for the detection of diverse selection signatures, including extended haplotype homozygosity (EHH) [11], the integrated haplotype score (iHS) [12], the composite of likelihood ratio (CLR) [13], the fixation index (Fst) [14], and others. A widely used method is the EHH test, which is based on linkage disequilibrium (LD) and haplotypes. It identifies selected genes by detecting long homozygous haplotype segments that form under positive selection, where the mutations and linked sites rapidly increase in frequency, creating extended LD. The EHH test captures this by measuring the extent of haplotype homozygosity over the distance to identify selected regions. The iHS test, an improvement on the EHH test, compares the EHH scores of ancestral and derived alleles at polymorphic loci. Under positive selection, the derived allele typically shows significantly higher EHH scores, indicating a selection signal. Moreover, the iHS mitigates the effect of recombination rate heterogeneity, making it more sensitive to positive selection signatures in moderate-frequency variants, thus effectively identifying selected gene regions.

With the rapid development of whole-genome resequencing technology and selection signal detection methods, we are now able to gain deeper insights into the selection mechanisms underlying the genetic improvement of Angus cattle. In this study, we employed the iHS test to analyze the selection signatures in 282 Angus cattle, focusing on genes associated with economic traits and their roles in immunity, reproduction, and meat quality. Through a comprehensive analysis of these signatures, we identified QTLs related to production, reproduction, and the meat and carcass, providing insights into the underlying genetic basis and biological significance of these QTLs. These findings provide a theoretical foundation for further genetic improvements and offer valuable guidance for optimizing the breeding strategies for Angus cattle in the Ningxia region.

## 2. Materials and Methods

### 2.1. Ethics Statement

All procedures involving cattle in this study were subjected to review and subsequent approval by the Animal Ethics Committee of the Institute of Animal Sciences, Chinese Academy of Agricultural Sciences (protocol code: IAS 2024-170) (Beijing, China).

### 2.2. Sample Collection and Sequencing

The group of cattle used in this study has its origins in the Angus core breeding population established by the Animal Husbandry Workstation of the Ningxia Autonomous Region. To ensure a broader representation of the Ningxia Angus population, we selected individuals based on the following criteria: both parents are purebred black Angus, have diverse sire origins, and individuals were selected to give a wide range of birth years. Specifically, the population included 271 offspring derived from 20 sires using frozen semen imported from the United States, Canada, Argentina, and Australia, as well as 11 additional frozen semen samples. Tail vein blood samples were collected from these 271 Angus cattle, and whole-genome resequencing was performed on 196 cows and 86 bulls. Genomic DNA was extracted from blood and tissue samples using the Magnetic Universal Genomic DNA kit (TIANGEN BIOTECH, Beijing, China). The integrity and contamination of the samples were evaluated through agarose gel electrophoresis. The total DNA concentration was determined using a Qubit 4.0 Fluorometer (Invitrogen, Carlsbad, CA, USA). Samples with a concentration exceeding 12.5 ng/μL and a mass greater than 1.0 μg were subjected to further analysis. A high-quality DNA library was constructed using the DNBSEQ-T7 sequencing platform from BGI in Wuhan, China. The read length for each individual was 2 × 150 bp, with an average of 387,596,237 reads and 58,139,435,494 bases.

### 2.3. Bioinformatics Analysis of Sequencing Data

To obtain high-quality data for the subsequent analyses, it was essential to perform quality control (QC) on the raw data. SOAPnuke (v.2.2.6) [15] was employed to eliminate low-quality raw reads containing adapters, an excess of Ns, or a large number of low-quality base reads. This was achieved through three filtering steps: (1) removing reads with more than 50% overlap with the adapter sequence, (2) eliminating reads with more than a 0.1% unknown base N content, and (3) discarding reads where more than 50% of their bases have a quality score of less than 10. Following the quality control, the resulting clean dataset comprised an average of 376,524,010 reads per individual, and an overall clean data rate of 97.14% across the population. Post-filtering, the proportion of bases with a quality score of 20 or above (Q20 (%)) was 97.78%, and the GC content of the DNA (GC (%)) was 43.90%. These QC steps produced higher-quality sequence data. The high proportion of Q20 (%) indicates not only a more accurate alignment of the reads to the reference genome but also improves the accuracy of detecting SNPs and other variations, thus providing a solid foundation for the subsequent analyses.

The clean data were aligned to the ARS-UCD1.2 reference genome using the mem algorithm of the BWA software (v.0.7.17) [16], and variants were detected using GATK (v.4.2.6.1) [17]. First, the HaplotypeCaller tool was employed to obtain the GVCF file for each sample, and then the CombineGVCFs tool was used to merge the GVCF files from all samples. Next, the GenotypeGVCFs tool was utilized to extract the variant loci from the GVCF files. Subsequently, SNP loci were extracted using the SelectVariants tool; this was followed by variant filtering with the VariantFiltration tool to exclude variants with a Quality by Depth (QD) < 2.0, Fisher’s Strand Test (FS) score > 60.0, Mapping Quality (MQ) < 40.0, MQRankSum < −12.5, and ReadPosRankSum < −8.0. To guarantee the precision and dependability of the downstream analyses, quality control was performed on the variant data using VCFtools (v.0.1.17) [18] and the following criteria: (1) exclude SNP loci located on sex chromosomes; (2) retain only bi-allelic loci (min-alleles: 2; max-alleles: 2); (3) exclude SNP loci with a missing rate greater than 10% (max-missing: 0.9); (4) exclude loci with a minor allele frequency (MAF) less than 5% (maf: 0.05); and (5) exclude loci that do not meet the Hardy–Weinberg equilibrium test P-value threshold of 1 × 10^−6^ (hwe: 0.000001).

### 2.4. Sequencing Data Annotation

The SNP loci that passed the QC were subsequently annotated using the SnpEff (v.5.1) software [19]. The genome reference sequence of ARS-UCD1.2 and the GFF3 annotation file were downloaded from the ENSEMBL website (https://ftp.ensembl.org/pub/release-110/gff3/bos_taurus/Bos_taurus.ARS-UCD1.2.110.gff3.gz, accessed on 16 July 2024). The SnpEff software was then used to construct the annotation database, and the information for the SNPs in the sequencing data was annotated and saved in the VCF file format. Next, the genomic annotation information from the VCF files was extracted using snpSift (v.5.1), and the variant annotations were classified into nine functional categories: intergenic region variants, downstream gene variants, 3′ UTR variants, 5′ UTR variants, intron variants, upstream gene variants, missense variants, synonymous variants, and other regulatory variants [20].

### 2.5. Selection Signatures Analysis

An iHS analysis was employed to identify the selection signatures present in the genomes of the Angus cattle populations in the Ningxia region after the genetic improvement process.

The formula for standardizing the iHSs using the norm module of the Selscan software (v.2.0.0) was as follows:

(1) $$iHS=\frac{ln(\frac{{iHH}_{A}}{{iHH}_{D}})-E_{p}[ln(\frac{{iHH}_{A}}{{iHH}_{D}})]}{{SD}_{p}[\frac{{iHH}_{A}}{{iHH}_{D}}]},$$

where *iHH_A_* and *iHH_D_* represent the integrated haplotype purity of the ancestral allele (A) and derived allele (D), respectively. $E_{p}[ln(\frac{{iHH}_{A}}{{iHH}_{D}})]$ and ${SD}_{p}[\frac{{iHH}_{A}}{{iHH}_{D}}]$ are the expectation and standard deviation of the frequency of bin *p*, respectively. The detection process set 800,000 bp as the maximum spacing, and the average iHS of the SNPs was calculated as the iHS of the selected regions in a 50 kb non-overlapping window covering the whole genome. Subsequently, based on the iHS of these regions, the regions with |iHS| values in the top 1% and with more than 10 SNPs were sePlease carefully check all the variables and parameters in the manuscript and keep them in the same format, italic or not.lected as the candidate selection regions.

Based on the gene annotation file of the ARS-UCD1.2 reference genome released by ENSEMBL (https://ftp.ensembl.org/pub/release-110/gtf/bos_taurus/Bos_taurus.ARS-UCD1.2.110.gtf.gz, accessed on 2 August 2024), the selected regions obtained from the aforementioned analysis were annotated using bedtools intersect in order to identify the potential candidate genes that were subjected to selection pressure within the population [21].

### 2.6. Candidate Gene Enrichment Analysis

In order to explore the biological functions of the candidate genes, this study used the g:Profiler website (https://biit.cs.ut.ee/gprofiler/gost, accessed on 2 August 2024) to perform Gene Ontology (GO) enrichment analysis, focusing on molecular functions (MFs), biological processes (BPs), and cellular components (CCs) [22]. The Bos taurus genome was used as the background in g:Profiler, and the results below the corrected threshold for g:Profiler Set Counts and Sizes (g:SCS) of <0.05 were considered significant. Further analysis of the GO enrichment results can reveal the biological characteristics that are closely related to the candidate genes, especially those related to the adaptive and economic traits of the Angus cattle population.

### 2.7. QTL Annotation and Enrichment Analysis

To further investigate the potential roles of the candidate selection regions in economic traits, we first downloaded cattle QTL information that was annotated based on the ARS-UCD1.2 reference genome from the Cattle QTL Database (release 53) (https://www.animalgenome.org/cgi-bin/QTLdb/BT/index, accessed on 2 August 2024). Then, using the R package GALLO, we conducted QTL annotation and enrichment analysis on the selection regions identified through the selection signature detection process to evaluate their enrichment across the different QTL categories. In the enrichment analysis, the correction threshold for hypothesis testing was set to FDR < 0.05 to ensure the significance of the enrichment results.

## 3. Results

### 3.1. Sequencing and SNP Calling

In the study, a total of 282 Angus cattle underwent whole-genome resequencing, with an average genome coverage depth of 20.69× (16.09–26.40×). The sequencing data were aligned to the ARS-UCD1.2 reference genome, with an average of 375,723,176 reads mapped per genome, covering 99.79% of the reference sequences (Table S1). After SNP calling and quality control, a total of 10,560,195 variant loci were identified for the subsequent analyses.

**Figure 1:**
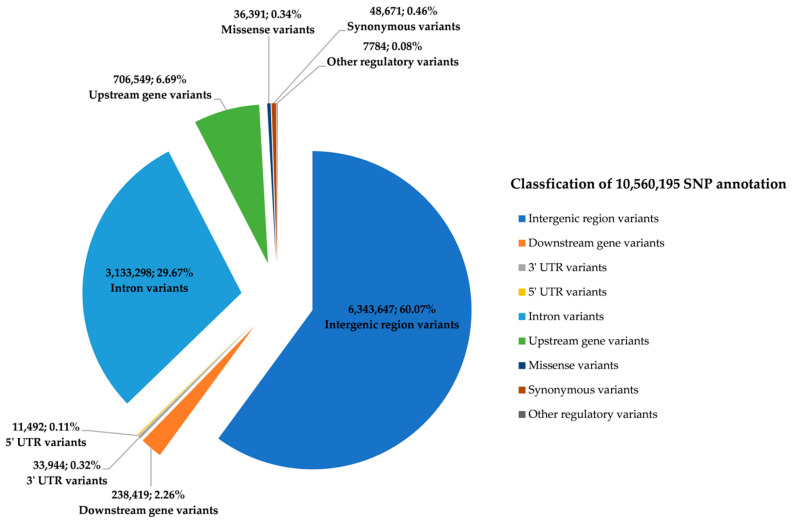
Annotation classification and distribution of resequencing variants.

The reference genome annotation database of ARS-UCD1.2.110 was constructed using SnpEff, and the 10,560,195 variant loci were categorized into 9 major groups (intergenic region variants, downstream gene variants, 3′ UTR variants, 5′ UTR variants, intron variants, upstream gene variants, missense variants, synonymous variants, and other regulatory variants) comprising 27 annotation types (Figure 1). The other regulatory variants had 16 annotations, including intragenic variant, splice region variant, start lost, and stop lost. Among all the annotated variants, intergenic region variants accounted for the largest proportion at 60.07%, followed by intron variants at 29.67%. The remaining seven variant types, in descending order of frequency, were upstream gene variants at 6.69%, downstream gene variants at 2.26%, synonymous variants at 0.46%, missense variants at 0.34%, 3′ UTR variants at 0.32%, 5′ UTR variants at 0.11%, and other regulatory variants at 0.07% (Table S2). This extensive dataset has significant value for advancing breeding and biological research in Angus cattle.

### 3.2. Selection Signatures and Gene Annotation in Selected Regions

**Figure 2:**
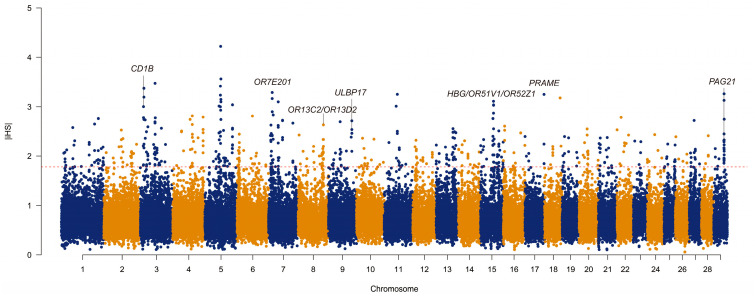
Distribution of genome-wide selection signatures.

In this study, the iHS method of the Selscan software was utilized to detect haplotype-based selection signatures, aiming to identify strong signatures of recent selection in the Ningxia Angus population. By calculating the mean iHSs of the SNPs within a 50 kb window and filtering out windows with less than 10 SNPs, a total of 49,562 50 kb windows were ultimately retained in this study. The top 1% of the 495 candidate regions with the highest absolute iHSs (|iHS| > 1.84) were selected (Figure 2). The highest |iHS| was located on chromosome 5 (chr5:58800001-58850001), with an |iHS| of 4.22, but there were no candidate genes that overlapped with this region. Gene annotation was performed on the 495 selected regions using bedtools, and finally, 300 candidate genes were annotated within 194 regions (Table S3), including apolipoprotein L3 (*APOL3*), UL16-binding protein 21 (*ULBP21*), tumor necrosis factor ligand superfamily member 11 (*TNFSF11*), family with sequence similarity 83 member D (*FAM83D*), CD1b molecule (*CD1B*), zinc finger protein 280B (*ZNF280B*), and olfactory receptor family 1 subfamily A member 1 (*OR1A1*), among others.

### 3.3. Functional Enrichment Analysis of the Candidate Genes

**Figure 3:**
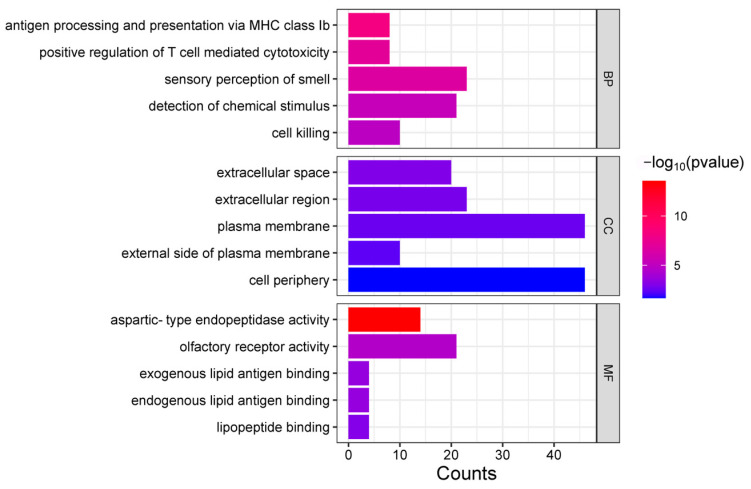
Enrichment analysis of GO of identified candidate genes.

The annotated candidate genes were subjected to enrichment analysis using the g:Profiler website to identify significantly enriched pathways or categories associated with specific biological processes, molecular functions, or cellular components. Setting the g:SCS threshold of <0.05 as the significance threshold, 59 significant GO terms were found to be enriched, including 9 molecular functions, 45 biological processes, and 5 cellular components (Table S4). We found that multiple GO terms were related to the regulation and response of the immune system, especially T cell-mediated immune responses and cytotoxic responses (Figure 3). The enriched GO terms included T cell-mediated cytotoxicity (GO:0001913), antigen processing and presentation (GO:0019882), and positive regulation of T cell-mediated immunity (GO:0002711), which are associated with immune system functions. Another significantly enriched category was associated with sensory perception functions, especially olfactory perception. Terms such as sensory perception of smell (GO:0007608), sensory perception of chemical stimulus (GO:0007606), and G protein-coupled receptor (GPCR) activity (GO:0004930) were found to be enriched, indicating a potential role of olfactory receptors (ORs) in the sensory perception functions of Angus cattle.

### 3.4. QTL Enrichment Analysis in Selected Regions

**Figure 4:**
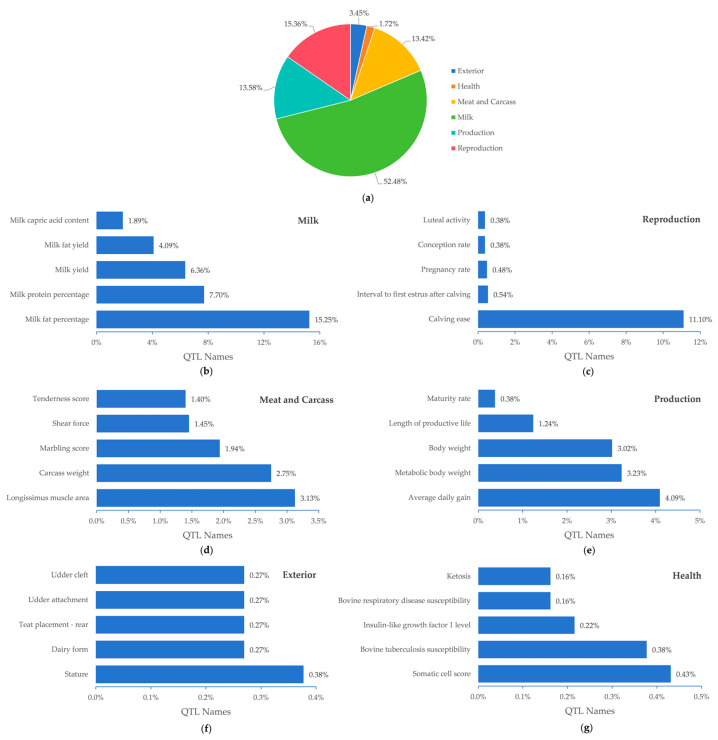
Proportional distribution of QTLs in selection signatures across trait categories. (**a**) Percentage of QTLs for different trait categories. (**b**–**g**) Proportion of QTLs for top five traits in the six trait categories.

QTL annotation of the selected segments was performed using the GALLO package. The QTL annotation results showed that the annotated segments related to exterior traits accounted for 3.45% of the QTLs, while those related to health, meat and carcass, milk, production, and reproduction traits accounted for 1.72%, 13.42%, 52.48%, 13.58%, and 15.36%, respectively (Figure 4a). Among the milk traits, the milk fat percentage accounted for the largest proportion, at 15.25% of the selected regions (Figure 4b). Notably, among the reproductive traits, calving ease overlapped with the selection signatures in a particularly large proportion of 11.1% (Figure 4c). Additionally, the longissimus muscle area under the meat and carcass traits and average daily gain, metabolic body weight, body weight under the production traits accounted for more than 3% of the selection segment (Figure 4d,e). Moreover, the detected selection signatures also covered QTLs for economically important traits such as stature, somatic cell score, and bovine tuberculosis susceptibility under the exterior and health traits (Figure 4f–g).

To assess the enrichment of QTLs associated with these six categories of economic traits in the selection segments, we performed enrichment analysis using the GALLO package. Using FDR < 0.05 as the threshold for screening, we obtained a total of 15 traits across 4 categories that were significantly enriched in the selection signatures, including the longissimus muscle area, carcass weight, and marbling score under the meat and carcass traits; milk fat percentage, milk lauric acid content, milk capric acid content, and 10 other milk traits; average daily gain under the production traits; and calving ease under the reproduction traits. The specific enrichment results are presented in Table 1.

## 4. Discussion

As one of the most popular beef cattle breeds in the world, Angus has been imported from the United States, Canada, and other countries to China due to its strong adaptability, low calving difficulty rate, good carcass quality, and distinct muscle marbling. Through the combined effects of natural and artificial selection, Angus cattle have adapted to different environmental conditions and production needs. The domestication process and selection pressure leave signals in the genome, which not only reflect the adaptation mechanism of Angus cattle, but they also show the selection direction for its economic traits. Therefore, detecting these selection signatures is crucial for understanding the phenotypic variation of complex traits and for identifying genes associated with economic traits in Angus cattle.

### 4.1. Selection of Immunity-Related Genes in the Genetic Improvement of Angus Cattle

In this study, a total of 495 candidate selection segments were identified by the iHS analysis using resequencing data from 282 Angus individuals. The gene annotation revealed 300 candidate genes across 194 selected segments, including nerve growth factor (*NGF*) (|iHS| = 2.02) [23], pregnancy-associated glycoprotein (*PAG*) family 1 (*PAG1*), *PAG4*, *PAG16*, *PAG19*, *PAG20*, and *PAG21* [24], and immune-related genes such as *ULBP21* (|iHS| = 2.17) [25], *CD1B* (|iHS| = 3.03) [26], and *TNFSF11* (|iHS| = 2.16). The GO enrichment analysis of all the annotated candidate genes identified 9 molecular functions, 45 biological processes, and 5 cellular components. Among the 45 terms, there were 30 pathways closely related to immune function, including antigen processing and presentation, regulation of lymphocyte-mediated immunity, positive regulation of adaptive immune responses, and others. We posit that the Angus cattle population may have experienced strong natural and artificial selection pressures on immune function during the breeding process, with immune-related genes and pathways playing a key role in both adaptive evolution and targeted breeding improvements.

Immune function in cattle is closely related to production, reproduction, milk, and other traits. Aleri et al. [27] previously demonstrated that calves with a high antibody-mediated immune response (AMIR) had a higher average daily gain compared to those with a low immune response. Additionally, Fleming et al. [28] found that colostrum from cows with a high AMIR had higher concentrations of both IgG and β-lactoglobulin (β-LG), indicating better colostrum quality. Higher IgG concentrations reduced the risk of failure in passive immunity transfer to calves, thereby providing better immune protection. High β-LG concentrations also contribute to preventing sepsis, diarrhea, and respiratory infections in calves. However, selection for a high milk yield can negatively impact immune function, affecting disease resistance and reproduction, for example, by lowering conception rates and reducing estrus visibility [29,30]. Therefore, balanced immune function is crucial not only for disease resistance in Angus cattle but also for enhancing herd performance and guiding effective genetic improvement.

The annotated genes *PAG1*, *PAG4*, *PAG19*, *PAG20*, and *PAG21* of the *PAG* gene family are all associated with three MFs: aspartic-type endopeptidase activity [31,32,33], aspartic-type peptidase activity, and endopeptidase activity. The *PAG* family encodes glycoproteins within the aspartic protease subclass, which optimally act as protein hydrolases under acidic pH conditions, aligning with their molecular function annotation of aspartic-type endopeptidase activity. PAG was first discovered in the placenta of pregnant cows in 1982 [34]. Subsequently, it was also found to be widely distributed in other mammals and closely linked to immune regulation during pregnancy and fetal development [35]. In immunomodulation, the major histocompatibility complex (MHC) is involved in the presentation of protein antigens. Roberts et al. [36] found that PAG competes with peptides for MHC binding, inhibiting T lymphocyte activation and exerting immunomodulatory effects at the maternal–fetal interface. Local immunosuppression is necessary to establish maternal–fetal histocompatibility during early pregnancy. Moreover, studies in cattle have linked PAG concentrations to monocyte and granulocyte activity, suggesting that PAG regulates maternal immune status, helping to protect the fetus from rejection [37,38]. Additionally, PAG is involved in the synthesis of pregnancy-related hormones. Prostaglandin E_2_ (PGE_2_) plays roles in preventing luteolysis and promoting luteinization, and as a luteinizing factor, PAG can stimulate the secretion of PGE_2_ in the placenta, thereby regulating progesterone synthesis in the corpus luteum to maintain pregnancy [39,40]. In conclusion, *PAG* family genes were selected for pregnancy-related immune regulation and are vital for enhancing reproductive success in Angus cattle.

*ULBP21* belongs to the *ULBP* family within the MHC Class I superfamily and, despite lacking the typical α3 domain, retains critical immune functions [41]. Retinoic acid early transcript 1G (*RAET1G*) is part of the similar *RAET1* family. In humans and mice, these genes encode transmembrane proteins that serve as stimulating ligands for the NKG2D receptor, thereby activating natural killer (NK) cells and T cells to enhance anti-tumor and anti-infection immunity [42,43,44]. The enrichment analysis revealed that *ULBP21* and *RAET1G* are significantly involved in 32 of the 45 biological processes, particularly in pathways related to MHC Class I and T cell-mediated immunity, such as antigen processing and presentation of endogenous peptide antigens via MHC Class Ib. These pathways rely on MHC Class I molecules to present endogenous antigens to CD8 T cells, triggering cytotoxic effects against infected and damaged cells [45,46]. As an activating receptor, NKG2D triggers NK cell cytotoxicity when bound to the proteins encoded by these two genes, aiding in the elimination of infected or abnormal cells [47,48]. Recent studies in cattle have shown that the *ULBP21* gene is located in variable regions associated with environmental resilience, which are closely linked to immune responses and resistance to external parasites [25,49]. Additionally, the *RAET1G* gene is also enriched in the natural killer cell lectin-like receptor-binding pathway [25]. These findings indicate that *ULBP21* and *RAET1G* may enhance disease resistance and adaptive immune responses in Angus cattle by modulating immune cell activity.

The *CD1B* gene, a member of the *CD1* gene family, encodes the CD1b protein, an antigen-presenting molecule. The enrichment analysis showed that *CD1B* is significantly enriched in 37 GO terms, primarily in lipid antigen presentation, MHC Class I, and T cell-mediated immunity. CD1b and MHC Class I molecules exhibit sequence homology and structural similarities, with CD1b primarily responsible for delivering lipid antigens to T cells, while MHC Class I molecules mainly present peptide antigens [50]. The CD1b protein induces T cell immune responses by binding and delivering lipid and glycolipid antigens. It endogenizes antigenic substances, encapsulating them in acidic vesicles before delivering them to the corresponding T cells. During endogenous delivery, the CD1b complex is phagocytosed by the MHC II complex (MIIC); in an acidic microenvironment, CD1b binds efficiently to recognizable exogenous lipid antigens to form stable structures that trigger immune responses [51,52,53,54]. Studies in cattle further demonstrated the critical role of CD1 molecules in presenting lipid and glycolipid antigens to T cells, which are essential for immune responses against Mycobacterium tuberculosis infections [55]. These findings underscore the importance of CD1-mediated lipid antigen presentation in combating intracellular bacterial infections. Consistent with this, the results from this study indicated that *CD1B* is enriched in MHC Class I related immune pathways, suggesting that Angus cattle may have experienced selection pressure on endogenous and exogenous lipid antigen presentation and T cell-mediated immune responses. This likely enhances their resistance to pathogens, particularly bacterial and parasitic infections.

The GO enrichment analysis showed that the *TNFSF11* gene is significantly enriched in immune response pathways, indicating its important role in the immune system of Angus cattle. The TNFSF11 protein encoded by this gene, also known as RANKL, is a type II transmembrane protein expressed on the surface of T lymphocytes, bone marrow mesenchymal stromal cells, osteoblasts, and other cells [56]. RANKL is essential for T and B cell development, and its deficiency leads to impaired B cell development in mice, which impedes the transformation from pro-B to pre-B cells [57]. In addition, studies have shown that RANKL may have distinct functions in various stages of the immune response. It has anti-inflammatory effects in the absence of inflammation but may promote T cell inflammation in chronic inflammatory environments, possibly due to increased expression on activated T cells [58,59,60]. Recent studies on foot-and-mouth disease virus (FMDV) in cattle have revealed that RANKL, as a key component of the non-canonical nuclear factor kappa-light-chain-enhancer of activated B cells (NF-κB) signaling pathway, is upregulated in transitional FMDV carriers. This suggests that the pathway may play a role in the establishment of persistent FMDV infections by promoting immune tolerance and suppressing the T helper 17 (Th17) cells response [61]. In addition, although *TNFSF11* was enriched only in the immune response pathway in this study, other research has highlighted its important function in bone metabolism. During bone remodeling, osteoclasts remove old or damaged bone, and osteoblasts form new bone [62]. RANKL is a key factor in osteoclast differentiation, activation, and survival. Dead osteoblasts release adenosine triphosphate (ATP) to stimulate RANKL expression in nearby osteoblasts [63]. As phagocytes cannot reach and phagocytose apoptotic osteoblasts are embedded in the bone matrix, these cells undergo secondary necrosis and release damage-associated molecular patterns (DAMPs), which promote RANKL-induced osteoclastogenesis [64]. The study also demonstrated that bovine RANKL (BoRANKL) can induce bone marrow cells to differentiate into osteoclasts in both cattle and sheep, showing cross-species reactivity [65,66]. Thus, *TNFSF11* is a key regulator of both the bovine immune system and bone metabolism. This dual function suggests that during adaptive selection in Angus cattle, this gene may be under selection pressures related to both immune function and bone health.

### 4.2. Genetic Selection Pressure and QTL Enrichment Analysis for Economic Traits in Angus Cattle

Selection signatures combined with QTL enrichment analysis can reveal the economic traits under significant selection pressure in Angus cattle during long-term breeding. Based on the Cattle QTL database, we found that approximately 52.48% of the signals were enriched in milk traits such as milk fat percentage, milk protein percentage, and milk yield, indicating that the milk quality and yield have been optimized in Angus cattle, with the relevant genes experiencing selection pressure. Studies have shown that milk yield during lactation correlates with calf weight and height. Calves with a shorter height tend to yield less milk in subsequent lactations, while medium-weight calves yield more during early lactation than heavier calves [67]. Although Angus cattle are primarily bred for beef, we hypothesize that milk production traits may indirectly affect their growth and calf health.

Calving ease is a notable advantage of Angus cattle, which helps reduce the difficulty of calving and subsequently enhances the health of both the dam and calf [68,69,70]. In this study, 15.36% of the QTL annotations were related to reproduction, with calving ease overlapping the selection regions by 11.1%, which was significantly higher than that of the other QTLs. Further enrichment analysis demonstrated that only calving ease was significantly enriched among the reproduction traits, aligning with the breeds’ low calving risk and indicating strong genetic selection pressure on this trait.

Excellent meat and carcass quality are additional favorable traits of Angus cattle [71]. In this study, the longissimus muscle area under meat and carcass traits, as well as average daily gain under the production traits, accounted for over 3% of the selection segments. The enrichment analysis highlighted four traits: longissimus muscle area, carcass weight, marbling score, and average daily gain. The marbling score is linked to the meat quality and flavor, while the longissimus muscle area, carcass weight, and daily gain affect the meat yield and production efficiency. These traits are crucial for a high breeding value and economic efficiency in beef production. These results suggest that genetic selection has significantly improved these key economic traits in Ningxia Angus cattle.

Although many immune-related genes were annotated by the selection signatures and enriched in immune pathways, only 1.72% of the QTLs were associated with health traits, much lower than those linked to milk, reproduction, production, and meat and carcass traits. This highlights an imbalance in the selection focus, where breeders prioritized economically profitable traits such as meat quality, growth rate, and milk production over health traits. While immune function is critical for disease resistance and overall herd health, immune-related traits had been overlooked at the QTL level. Consequently, although immune-related genes contribute to the natural adaptability and survival of Angus cattle, they have not been directly targeted in breeding efforts, potentially leaving them less optimized for long-term health and disease resistance. Nevertheless, immune function improvements may have interactive effects with other economically profitable traits. For instance, certain immune-related genes could positively impact the overall health of Angus cattle, which indirectly enhances traits like meat yield and reproductive success. Therefore, although there were few health-related QTLs, selection pressures on immune-related genes may still indirectly enhance production traits, reflecting their underlying role in supporting overall economic performance. This emphasizes the critical importance of balancing the selection of economic and health traits in future breeding programs to ensure the sustainable improvement of Angus cattle.

## 5. Conclusions

Here, we constructed a high-quality genomic variation dataset using whole-genome resequencing data from 282 Angus cattle and identified 300 positive selection loci, including the immune-related genes *PAG1*, *PAG4*, *PAG19*, *PAG20*, and *PAG21* of the *PAG* family, as well as *ULBP21*, *RAET1G*, *CD1B*, and *TNFSF11*. Additionally, through the QTL enrichment analysis, we also found that Angus cattle are under significant genetic selection pressure for economic traits such as reproduction and meat and carcass traits. These results suggest that Angus cattle not only have improved adaptive immune function but also made significant progress in production performance and reproductive capabilities during long-term selective breeding. This also provides a theoretical basis for further research into the molecular mechanisms and genetic diversity of important traits in Angus cattle.

## Figures and Tables

**Table 1:** Enrichment analysis results of selection signatures in all classes of trait QTLs.

Trait	Richness Factor	FDR	QTL Type
Longissimus muscle area	3.59	9.47 × 10^−15^	Meat and Carcass
Carcass weight	2.22	3.84 × 10^−6^	Meat and Carcass
Marbling score	1.74	1.04 × 10^−2^	Meat and Carcass
Milk fat percentage	2.27	3.17 × 10^−36^	Milk
Milk lauric acid content	5.29	1.37 × 10^−8^	Milk
Milk capric acid content	3.38	2.37 × 10^−8^	Milk
Milk yield	1.61	6.65 × 10^−6^	Milk
Milk fat-to-protein ratio	17.02	1.76 × 10^−5^	Milk
Milk caprylic acid content	2.71	1.33 × 10^−4^	Milk
Milk protein percentage	1.43	1.93 × 10^−4^	Milk
Milk caproic acid content	2.74	4.77 × 10^−4^	Milk
Lactation persistency	4.19	7.96 × 10^−4^	Milk
Milk linolenic acid content	2.52	5.24 × 10^−3^	Milk
Average daily gain	2.13	3.24 × 10^−8^	Production
Calving ease	4.74	1.43 × 10^−73^	Reproduction

## Data Availability

All whole-genome resequencing data have been submitted to the Sequence Read Archive (SRA) under accession number PRJNA1191236. The data will be available after 25 November 2028.

## Supplementary Materials

The following supporting information can be downloaded at: https://www.mdpi.com/article/10.3390/ani15010058/s1, Table S1: Alignment and coverage statistics of whole-genome resequencing data from 282 Angus cattle; Table S2: Annotation classification and statistical information for the 10,560,195 resequencing variants; Table S3: The 300 annotated candidate genes in the selection regions; Table S4: Results of GO enrichment analysis of candidate genes in selection signatures.
